# Commentary: Construction and validation of prognostic signatures related to mitochondria and macrophage polarization in gastric cancer

**DOI:** 10.3389/fonc.2024.1494136

**Published:** 2024-11-14

**Authors:** Bingdi Wei, Zhe Zhao, Yaohui Chen, Yonghong Li

**Affiliations:** Gansu University of Chinese Medicine, Lanzhou, China

**Keywords:** gastric cancer, mitochondria, macrophage polarization, single-cell data, prognostic signature

## 1 Introduction

Gastric cancer is a complex and highly prevalent malignant tumor with a multifaceted etiology. Its early symptoms are not obvious, and most cases are diagnosed at mid to late stages, limiting treatment effectiveness. Although surgery, chemotherapy, and emerging targeted therapies and immunotherapies have made some progress, early diagnosis and effective treatment remain challenging, highlighting the urgent need to explore new therapeutic approaches (1–3). Mitochondrial dysfunction and macrophage polarization play key roles in the occurrence and development of gastric cancer, with a close interaction between them (4–7). Mitochondrial abnormalities can promote macrophage polarization, and polarized macrophages can further influence the mitochondrial function of tumor cells (8). Currently, there are limited reports on the relationship between mitochondrial and macrophage polarization-related genes in tumors, and even fewer studies focus on the prognostic genes associated with these functions in gastric cancer and their potential molecular mechanisms. Zhang et al. identified gastric cancer prognostic genes related to mitochondrial function and macrophage polarization through data analysis, constructed a prognostic model, and explored the biological pathways of these genes and their relationship with clinical features (9). This research provides new insights into the pathological mechanisms of gastric cancer and its treatment. The study stands out for its innovation and data analysis but requires improvement in biological validation and sample diversity.

## 2 Commentary and discussion

This study pioneered a novel approach by linking mitochondrial function and macrophage polarization, two critical biological processes, to gastric cancer prognosis. Unlike most previous research, which focused on individual genes or pathways, this study integrates two processes closely related to tumor metabolism and the immune microenvironment, providing a more comprehensive prognostic model. Such multidimensional analysis is rare in gastric cancer research and opens new avenues for personalized treatment.

Methodologically, the study employed various bioinformatics tools, including Weighted Gene Co-expression Network Analysis (WGCNA), Cox regression analysis, and LASSO regression modeling, ensuring the scientific rigor of gene selection and the robustness of the prognostic model. Additionally, the study validated its findings using multiple datasets (TCGA and GEO), enhancing the model’s generalizability and applicability. By conducting cross-validation across different datasets, the authors ensured the reliability and predictive power of their results.

In terms of data acquisition, Zhang and colleagues utilized several authoritative public databases, such as TCGA and GEO, which provided large sample sizes and rich clinical data, ensuring the representativeness and accuracy of the analysis. The use of multicenter data further increased the credibility of the findings, laying a solid foundation for clinical applications.

However, despite analyzing multiple datasets, all data were derived from public databases, most of which involved Western populations. The study lacks analysis of patients from different ethnicities or regions, which could limit the model’s generalizability. Particularly, gastric cancer is highly prevalent in Asia, but the study does not include specific analyses of Asian patients (10). Moreover, the biological validation, although it performed qRT-PCR on key genes, was limited to 10 clinical samples, an insufficient number to support broad clinical application. In clinical research, sample size is a critical determinant of the validity and reliability of study findings. A small sample size diminishes the probability that statistically significant results will accurately reflect the true underlying effect (11). Even when statistical significance is achieved, studies with insufficient sample sizes are more prone to generating misleading or false-positive results. Consequently, conclusions derived from small sample studies should be interpreted with caution, as they often lack the robustness needed for reliable inference (12). In many research contexts, a minimum sample size of 30 participants per group is often considered a baseline to achieve approximate normality and statistical reliability, especially in small-scale experimental designs (13).

Besides, The study primarily focuses on predictive data analysis, with limited exploration of the underlying biological mechanisms. Future research should further investigate the specific functions and regulatory pathways of these genes. While the study highlights the importance of mitochondrial function and macrophage polarization in gastric cancer, it does not delve deeply into the mechanisms by which these processes influence tumor progression. Although the model shows good predictive capability, the lack of mechanistic insight may hinder its application in targeted therapy.

Moreover, the study’s lack of longitudinal data is a significant limitation, hindering a comprehensive understanding of the temporal dynamics of gene expression and their impact on disease progression in gastric cancer. Longitudinal studies are pivotal for tracking how mitochondrial function and macrophage polarization evolve over time, offering valuable insights into their mechanistic contributions to tumor development and metastasis. Without such time-sensitive data, assessing the long-term predictive accuracy of the model and its relevance to patient outcomes remains challenging. Incorporating longitudinal research would not only enhance the ability to monitor changes in gene regulation and immune responses but also reveal key regulatory pathways and interactions between the tumor microenvironment and cancer cells that remain elusive in cross-sectional analyses. This comprehensive approach could lead to the identification of biomarkers for early detection or new therapeutic targets, thereby improving the model’s potential for application in personalized and targeted therapies. Furthermore, longitudinal studies could validate predictive models by correlating mechanistic insights with observed clinical outcomes, advancing both the theoretical and practical applications of these findings in gastric cancer research.

By integrating mitochondrial function and macrophage polarization into a prognostic model for gastric cancer, this study offers a fresh perspective and provides a theoretical foundation for future research into tumor metabolism and immune regulation. The findings deepen our understanding of the tumor microenvironment in gastric cancer and encourage further investigation into these processes, particularly through longitudinal studies that capture temporal changes in gene expression and immune responses. If validated in larger, multicenter clinical samples enriched with longitudinal data, this prognostic model could become a valuable tool for clinical decision-making. Quantifying patients’ risk scores would enable clinicians to offer more personalized treatment plans for high-risk patients, reducing uncertainty in the treatment process.

To improve the broader applicability and clinical value of this research, several areas could be addressed in future studies. First, expanding the sample size, particularly in the clinical validation phase, to include patients from different ethnicities and regions would improve the model’s generalizability. Additionally, conducting longitudinal studies is essential to understand the temporal dynamics of gene expression and their impact on disease progression. Further exploration of the biological functions of key genes and their specific roles in gastric cancer is also necessary. More *in vivo* and *in vitro* experiments are needed to confirm these genes’ roles in tumor progression. Moreover, integrating multi-omics data, such as genomics, epigenomics, and proteomics, could further enhance the model’s predictive accuracy and reliability. Finally, if successfully validated, clinical applications could be developed, such as tools for detecting key gene expression levels in blood or tissue samples to calculate patients’ risk scores, offering more precise guidance for clinical treatment decisions.

In summary, this study innovatively combines mitochondrial function and macrophage polarization to construct a prognostic model for gastric cancer, offering a new research perspective. Its methodological rigor and reliable data analysis reflect strong theoretical and clinical potential. However, limitations remain in sample diversity, the absence of longitudinal data, and biological validation. Future research should address these issues by expanding clinical validation to more diverse populations, incorporating longitudinal studies, and conducting deeper mechanistic investigations. This approach would enhance the model’s robustness and applicability, ultimately improving clinical outcomes for patients.
